# Thioredoxin/Txnip: Redoxisome, as a Redox Switch for the Pathogenesis of Diseases

**DOI:** 10.3389/fimmu.2013.00514

**Published:** 2014-01-09

**Authors:** Eiji Yoshihara, So Masaki, Yoshiyuki Matsuo, Zhe Chen, Hai Tian, Junji Yodoi

**Affiliations:** ^1^Institute for Virus Research, Kyoto University, Kyoto, Japan; ^2^Advanced Chemical Technology Center in Kyoto (ACT Kyoto), JBPA Research Institute, Kyoto, Japan; ^3^Redox Bio Science Inc., Kyoto, Japan

**Keywords:** thioredoxin, Txnip, redoxisome, inflammation, diabetes mellitus, redox regulation

## Abstract

During the past few decades, it has been widely recognized that Reduction-Oxidation (redox) responses occurring at the intra- and extra-cellular levels are one of most important biological phenomena and dysregulated redox responses are involved in the initiation and progression of multiple diseases. Thioredoxin1 (Trx1) and Thioredoxin2 (Trx2), mainly located in the cytoplasm and mitochondria, respectively, are ubiquitously expressed in variety of cells and control cellular reactive oxygen species by reducing the disulfides into thiol groups. Thioredoxin interacting protein (Txnip/thioredoxin binding protein-2/vitamin D3 upregulated protein) directly binds to Trx1 and Trx2 (Trx) and inhibit the reducing activity of Trx through their disulfide exchange. Recent studies have revealed that Trx1 and Txnip are involved in some critical redox-dependent signal pathways including NLRP-3 inflammasome activation in a redox-dependent manner. Therefore, Trx/Txnip, a redox-sensitive signaling complex is a regulator of cellular redox status and has emerged as a key component in the link between redox regulation and the pathogenesis of diseases. Here, we review the novel functional concept of the redox-related protein complex, named “Redoxisome,” consisting of Trx/Txnip, as a critical regulator for intra- and extra-cellular redox signaling, involved in the pathogenesis of various diseases such as cancer, autoimmune disease, and diabetes.

## Introduction

The thioredoxin (Trx) system, composed of NADPH, thioredoxin reductase (TrxR), and Trx, is a key antioxidant system that protects cells from oxidative stress through its disulfide reductase activity. Trx1 and Trx2 are mainly localized in the cytoplasm and the mitochondria respectively, suggestive of their specific roles at different cellular compartments (Box 1). Trx are highly conserved in many organisms ranging from bacterial organisms to plants and mammals, indicating that the Trx system is a cellular system, essential for cellular survival and function. Recent studies have shown that an important Trx binding protein, thioredoxin interacting protein [Txnip/thioredoxin binding protein-2 (TBP-2)/vitamin D3 upregulated protein (VDUP1)] has the reciprocal function with Trx in the pathogenesis of disease such as autoimmune disease, cancer, and diabetes (1–3). Decades of research have implicated Trx/Txnip regulation as an attractive therapeutic target. Here we review the novel protein signaling complex we call “Redoxisome” as a redox-related signal transducer to highlight the novel therapeutic approach by Trx/Txnip.

Box 1 **Localization of Trx and Txnip, predicted existence of redoxisome in nucleus, cytosol, mitochondria, cellular membrane, and extra-cellular space.**

It is well known that Trx1 is localized in the cytosol, plasma membrane (PM), and nucleus as well as the extra-cellular space. Since Trx2 is localized in only mitochondria, the Trx/Txnip redoxisome system is mainly works as the Trx2/Txnip complex in mitochondria. It was found that this complex only occurs under the oxidative stress since Txnip is shuttled into mitochondria under stress and remains in the nucleus under the normal condition (4). Recently, it was found that Txnip is also located in PM (5). Poly-ADP-ribose polymerase 1 (Parp1) was found to be a binding protein for Txnip in the nucleus and the inhibition of Parp1 increases PM associated Txnip localization in human umbilical vein endothelial cells (HUVECs), suggesting that the translocation of Txnip from the nucleus to the PM is exclusively related to Parp1 inhibition. Since Txnip and Parp1 are both regulated by changes in cellular redox state in HUVECs, this novel PM associated Txnip could be associated with Trx1 and form a redoxisome system in the PM. Nuclear transport protein, importin-α was identified as a binding protein of Txnip, and the binding leads to the translocation of Txnip from the cytosol to the nucleus (6). Although it is still unknown whether Txnip is released into the extra-cellular space like Trx1, further studies about the localization specific Trx/Txnip, redoxisome system could give us a novel insight for a redox-dependent biological function in the cells.

## Thioredoxin1 and Thioredoxin2

In mammalian cells there are two isoforms of Trx called as Thioredoxin1 (Trx1) and Thioredoxin2 (Trx2) (7). Trx1 is mainly located in cytosol but also translocate to the nucleus and can be secreted from cells under certain circumstances [circulatory thioredoxin function were reviewed by our recent paper (8)], whereas Trx2 is located only in mitochondria. Trx1 is a 12-kDa ubiquitous protein that has disulfide-reducing activity. Trx are characterized by the presence of three conserved prolines, with one located between the catalytic cysteine residues of the – Cys-Gly-Pro-Cys – motif. Two cysteine residues (Cys-32 and -35) of the active site – Cys-Gly-Pro-Cys – are responsible for this reducing activity. This proline is the key residue that determines the reducing power of Trx and replacing it by a serine or a threonine has a dramatic effect on the redox and stability properties of the protein (9–13). Trx1 was originally identified as a hydrogen donor for ribonucleotide reductase in *Escherichia coli* (14). We have identified human Trx1 as an adult T cell leukemia-derived factor (ADF) from the supernatant of human T-cell leukemia type-1 (HTLV-1) infected T cell line, which was initially defined as autoimmune lymphokine and IL-2 receptor-inducing factor (15). In line with its function to respond to oxidative stresses, Trx1 expression is induced by variety of physiochemical stimuli, including virus infection, mitogen, UV-irradiation, hydrogen peroxide, ischemia reperfusion, which we have broadly reviewed (1, 3, 8, 16). The crystal structures of Trx1 in both oxidized and reduced states have been resolved and revealed that Trx1 has a basic Trx-fold (consisting of four β_2_-strands surrounded by three α_2_-helices) with additional α_2_-helices and β_2_-strands at the N-terminus (10, 17). Natural metabolic or endocrine substances including hemin, estrogen, prostaglandins, sulforaphane, and cAMP can also induce the expression and secretion of Trx1. A series of stress-responsive elements in the promoter region have been identified, including the oxidative stress response element (ORE), antioxidant responsive element (ARE), cAMP responsive element (CRE), xenobiotics responsive element (XRE), and Sp-1. Trx1 is also induced by fragrant unsaturated aldehydes from edible plants, through their ARE in the promoter region, meaning that Trx1 may be beneficial for protection against oxidative stress-induced cellular damage (18). Trx1-knockout mice are lethal due to early development and morphogenesis failure of the mouse embryo (19), whereas Trx1-transgenic (Trx1 Tg) mice are more resistant to oxidative stress with longer life span compared with wild type mice (20). These results suggest that Trx1 is an essential molecule for cellular and organismal survival. Decades of research have shown that Trx1 binds and modulates together proteins such as Nuclear Factor-κB (NF-κB), p53, hypoxia inducible factor-1 (HIF-1), Forkhead box class O1 (Foxo1), glucocorticoid receptor (GR), and Estrogen Receptor (ER) by a thiol-disulfide exchange reaction (3, 21). Trx1 binding protein that we identified in 1999 and named TBP-2, now commonly called as Txnip is a unique Trx binding protein that has a role as an endogenous inhibitor of Trx, since Txnip binding to Trx inhibits. Their protein reducing activity and/or Trx expression (22). This finding has implicated that redox-sensitive proteins and related cellular processes such as metabolism, proliferation, and inflammation could be regulated by Trx/Txnip signaling.

## Thioredoxin Interacting Protein (TBP-2/VDUP1)

Thioredoxin interacting protein (TBP-2/VDUP1) was originally cloned as a vitamin D3 target gene in HL-60 cells (23) (named as VDUP1). This molecule has emerged as a key component of cellular redox regulation since it was identified as binding partner of Trx and suggested to be an endogenous Trx inhibitor (22) (named as TBP-2). It has been known that the two Txnip cysteins are important for thioredoxin binding through a disulfide exchange reaction between oxidized Txnip and reduced Trx (24). This clear evidence suggests that the Trx-Txnip complex is important for redox-dependent cell function. Interestingly, Txnip is a member of the α-arrestin protein family (Arrdc1–5 and Txnip) containing two characteristic arrestin-like domains with PxxP sequence, which is a known binding motif for SH3-domains containing proteins, and PPxY sequence that is know binding motifs for WW-domain (2, 24–27). Since Txnip has a specific arrestin-like domain, which is responsible for protein–protein binding, many interacting protein for Txnip have been identified such as importin-α, transcriptional co-repressors SMRT-mSin3-HDAC (histone deacetylase), Jab1, E3 ubiquitinligase itch, Mybbp1a, and NOD-like receptor Protein-3 (NLRP3) as well as Trx (6, 28–32). These finding raises the possibility that Txnip may have a role for scaffolding in a signaling complex. Interestingly, genetic mapping identified a nonsense mutation in the Txnip gene as the cause for the phenotype in the Hcb-19 mutant mouse, which resembles familial combined hyperlipidemia (FCHL) (named as Txnip) (33). The mutation causes the truncation of Txnip in a critical region, which was reported to mediate Txnip’s binding to Trx1. Moreover Hcb-19 mice have decreased CO_2_ production but increased ketone body synthesis, suggesting that altered redox status may have a role in lipid metabolism such as citric-acid cycle and fatty acid utilization (34).

We reported that disruption of Txnip (Txnip KO) by gene targeting in mice causes predisposition to death with severe bleeding, hypoglycemia, hyperinsulinemia, and liver steatosis during fasting (35). Txnip gene expression is induced in fasting, and the key transcription regulator peroxisome proliferator activated receptor-alpha (PPAR-α) and sterol response element binging protein (SREBP) signaling are dysregulated in the liver of Txnip KO during the feeding-fasting nutritional transition (36). Txnip expression is widely regulated by nutritional status, signal, and enzyme such as feeding-fasting, obesity, high glucose, amino acids, nuclear receptor signal (PPARs, VDR, GR), and AMPK (23, 26, 28, 36–41). These results clearly suggest that Txnip is an important molecule that regulates glucose and lipid homeostasis.

A critical role of Trx and Txnip in inflammation, cancer progression, and diabetes is mentioned in a later part of this review.

## Thioredoxin1/Txnip, Redoxisome, a Redox-Related Signal Complex

**Figure 1:**
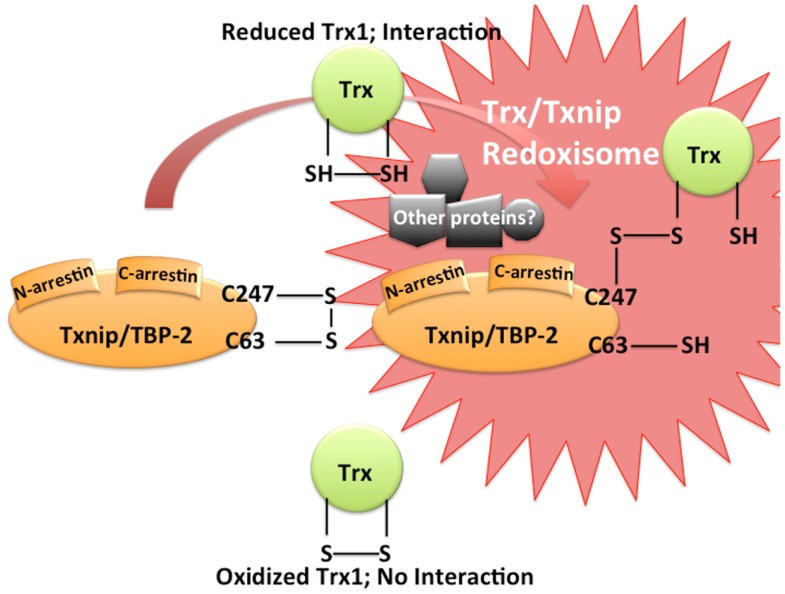
**Trx/Txnip signaling complex as redoxisome**. Txnip contains an intramolecular disulfide band between Cys-63 and -247 that allows efficient interaction with Trx. Txnip forms disulfide bond with reduced TRX by disulfide exchange, making a stable Trx mixed disulfide.

**Figure 2:**
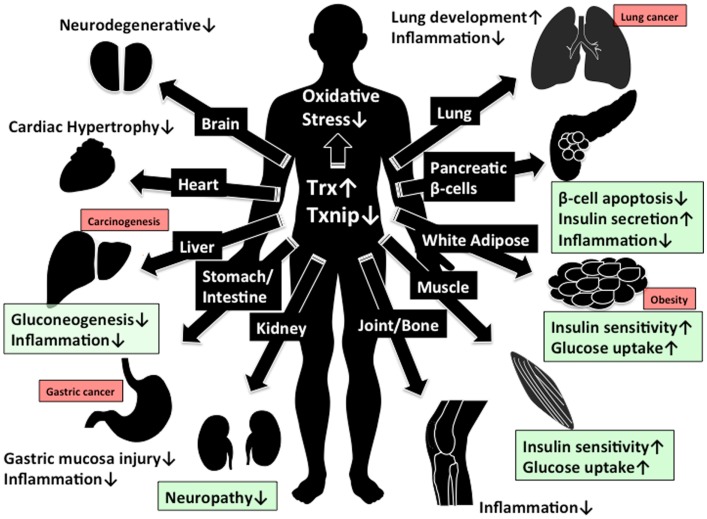
**Beneficial effect of Trx1/Txnip signaling for clinical aspect**. The beneficial effect by increasing of Trx1 and decreasing of Txnip expression are shown. Green box indicate the beneficial effect in diabetes while red box indicate the concern about adverse effect in cancer development by reduced Txnip expression.

The Trx system plays an important role in maintaining a reduced environment in the cell. We first identified Txnip/TBP-2/VDUP1 as an endogenous Trx1 binding and inhibiting protein (22). Interestingly, Txnip binds to reduced Trx1 but not to oxidized Trx1 nor to mutant Trx1, in which two redox active cysteine residues are substituted by serine (22). Since the disulfide exchange reaction between oxidized Txnip and reduced Trx1 [Txnip and Trxs form a stable disulfide-linked complex (24)] is known as the essential event for the interaction between Txnip and Trx1, two Txnip cysteines are important for Trx1 binding (Figure 1). These cysteines are not conserved in the broader family of arrestin domain-containing proteins, therefore, the Trx1 binding property of Txnip is unique (24). Thus, the catalytic center of Trx1 seems to be important for the interaction. This interaction is important for cellular redox regulation since the protein reducing activity of Trx1 is actually inhibited by Txnip interaction (22, 24). In COS-7 and HEK293 cells transiently transfected with Txnip expression vector, decreased of insulin reducing activity of Trx1 and diminished expression of Trx1 was observed (22). In addition, treatment of HL-60 cells with 1α, 25-dihydroxyvitamin D_3_ caused an increase in Txnip expression and down-regulation of the expression and the reducing activity of Trx1. These results suggest that Txnip serves as a negative regulator of the biological function and expression of Trx1 by direct interaction and provides insight into the redox-dependent signal complex (Figure 1). We would like to propose the concept of “Redoxisome” that the signaling complex, composed with Trx and Txnip as a redox-dependent signal complexes, “Redoxisome” since it seems this signal complex could be key regulatory mechanism in multiple condition and diseases (Figure 2).

## Thioredoxin2/Txnip, Redoxisome in Mitochondria

The Trx/Txnip, redoxisome system also exists in mitochondria (42–44). The major difference between the cytosolic Trx system and the mitochondrial Trx system is that cytosolic Trx system consists of Trx1, TrxR, and Peroxiredoxin (Prx) while that of mitochondria consists of Trx2, TrxR2, and Prx3 (45, 46). Interestingly, Txnip interacts with both cytosolic Trx1 and mitochondrial Trx2 (4, 47), meaning that the Trx/Txnip, redoxisome system works in both cytosol and mitochondria. Txnip can translocate to the mitochondria, where it binds to oxidized Trx2 leading to mitochondrial dysfunction (4). Since Trx2 bind to Apoptosis signal regulating kinase 1 (Ask1), a major mitogen-activated protein kinase kinase kinase (MAPKKK) and inhibits Ask1 phosphorylation and activation, increased binding of Txnip to Trx2 reduces the interaction between Trx2 and Ask1 and induces Ask1 activation for apoptosis (4). It is also reported that Txnip translocation increase reactive oxygen species (ROS) accumulation in mitochondria and leads to activation of the NLRP3 inflammasome (47). However the function of Trx2 in regulating the inflammasome has not been studied to determine whether it is an essential component of NLRP3 activation in mitochondria.

## Thioredoxin1/Txnip, Redoxisome, Critical for Pathogenesis of Type 1 and 2 Diabetes

Diabetes is characterized by high blood glucose levels, as results of insufficient insulin due to reduced insulin secretion and/or insulin sensitivity for the body’s required. Type 1 Diabetes (T1DM) is an autoimmune disease that results in β-cells destruction. It usually presents in childhood, accounts for 5–10% of all diabetes, and is associated with the presence of islet-cell antibodies, and patients require lifelong insulin treatment. While, Type 2 Diabetes (T2DM), the most common form of the disease, is characterized by defects in both insulin secretion from pancreatic β-cells and insulin sensitivity in peripheral tissues such as skeletal muscle, liver, and fat. T2DM is influenced by life style factors such as age, pregnancy, and obesity, but also has a strong genetic component (48). Despite the differential mechanism for the pathogenesis of T1DM and T2DM, oxidative stress is commonly related to the pathogenesis of the disease (49). It has been known that low levels of cellular ROS are required for cellular signaling, such as optimal tyrosine phosphorylation-dependent signaling *in vitro* (49–51), while chronic excessive generation of ROS aggravate insulin sensitivity in skeletal muscle and disrupt β-cell function and survival (52), suggesting that an optimal balance of cellular redox regulation is critical for the pathogenesis of both T1DM and T2DM.

Thioredoxin1-transgenic mice on a C57BL/6J background, in which human Trx1 is systemically overexpressed under control of the β-actin promoter, are more resistant to various oxidative stresses than control mice (20, 53–57). Trx1 Tg mice have more resistance to develop both T1DM and T2DM in mice (55–58). In non-obese diabetic (NOD) mice (T1DM model) or streptozocin (STZ) induced T1DM model mice, β-cell-specific overexpression of Trx1 markedly reduces the incidence of diabetes without improving insulin secretory capacity, insulin content, or the development of insulitis compared with those of littermate controls (55).

Recently it was reported that in an animal model of T2DM, obese diabetic db/db mice, β-cell-specific Trx1 overexpression suppresses progressive β-cell failure (56). Trx1 Tg db/db mice exhibit significantly lower blood glucose levels and higher plasma insulin levels compared with littermate controls (56). These results suggest that Trx1 has a protective effect on reducing oxidative stress inducing failure in T1DM and T2DM.

The endogenous Trx1 inhibitor, Txnip, was implicated as a redox rheostat to control Trx1 activity and expression. Recent studies suggested that Txnip expression is increased in skeletal muscle of human T2DM and impaired glucose tolerance (IGT) patients (59, 60). It was also reported that a genetic variation of the Txnip gene is associated with hypertriglyceridemia and increased diastolic blood pressure (61). Interestingly, Txnip expression is induced by high glucose conditions, while it is reduced by insulin (59). We reported that Txnip is more abundantly expressed in pancreatic islets, skeletal muscle, adipose, and kidney of leptin deficient ob/ob mice (T2DM model) compared with WT control mice and Txnip deletion in ob/ob mice (T2DM model) dramatically improves insulin resistance in skeletal muscle and β-cell insulin secretion function and survival (28). Piled evidence suggested that Txnip induced β-cell apoptosis under various kinds of stresses such as STZ treatment (T1DM model) (62, 63), high glucose (28, 29, 64, 65), ER-stress (66–68), dexamethasone/glucocorticoid (69), and inflammation/cytokine (29, 62), while it has been reported that Txnip aggravates hepatic glucose production (70) and insulin sensitivity in skeletal muscle (28, 71) and adipose tissues (72, 73). These evidences suggest that Trx1 and Txnip have an antagonistic function in progression of both T1DM and T2DM.

## Thioredoxin1/Txnip, Redoxisome, a Novel Regulator of NLRP3 Inflammasome and Inflammation

Human Trx1 is originally identified as an ADF (15, 74). Numerous evidences suggest that Trx1 is an anti-inflammatory molecule in both an intracellular and extra-cellular (8, 75–79) environment. Interestingly Trx is highly expressed in human T cell leukemia virus type-1 (HTLV-1)-transformed cell lines (ATL model cell line), whereas the Txnip expression is lost in HTLV-I-positive, interleukin-2-independent T cell lines but not in HTLV-I-negative T cell lines (37, 80, 81), suggesting a role for Trx1/Txnip in virus infection, prevention, and inflammation. Indeed, recent works revealed that Txnip has important role in inflammation in response to excessive nutrition, oxidative stress, and lipopolysaccharide (LPS) stimulation (29, 66–68, 82–85).

Recent publications suggest a physical interaction between Txnip and NLRP3, a key component of NLRP3 inflammasome (29, 66, 67, 82, 83). The inflammasome is the multiprotein complex that controls the activation of caspase-1 in the innate immune system that result in maturation of IL-1β. ROS is the major activator of the NLRP3 inflammasome. The physical interaction between Txnip and NLRP3 may explain the inflammasome activation in a ROS-sensitive manner (29) especially in macrophages in pancreatic islets (29, 67). The study, performed by Zhou et al. suggested that under the unstressed condition, Txnip is bound to Trx1 and NLRP-3 inflammasome is in active because of an absence of Txnip interaction with NLRP3, while under the oxidative stress condition, ROS generation facilitates Trx1-Txnip dissociation, thus increasing NLRP3-Txnip interaction. However there are some conflicting reports. Although Koenen et al. demonstrate that hyperglycemia activates caspase-1 and Txnip, glucose-induced activation of Txnip mediates an increase of IL-1β mRNA and intracellular pro-IL-1β rather than IL-1β processing (73). Furthermore, our collaborator Masters et al. and we found no significant difference in IL-1β secretion in response to inflammasome activators in bone marrow derived macrophages of Txnip KO mice (86). These evidences imply that the redox-dependent NLRP3 inflammasome activation is performed not only by Txnip-NLRP3 direct interaction but also by other mechanisms regulated by Trx1/Txnip with other participants for redoxisome signaling. Thioredoxin reductase (TrxR) might be involved in redox-sensitive inflammasome activation through Trx/Txnip, redoxisome signal since it has reported that under the absence of TrxR condition, Trx1 is able to recycle substrates at the expense of an alternative electron donor that is required to be oxidized form of Trx1 (87). It is possible there is an unknown potential redoxisome signal including TrxR, Trx1, and Txnip that lead to the activation of NLRP3 inflammasome.

Although increased production of ROS is often though to be implicated in the activation of inflammation, redox-related signal molecules are involved in NLRP3 inflammasome activation in a ROS-independent manner. It has reported that cells from patients with chronic granulomatous disease (CGD), characterized by an incapacity of phagocytosis to produce ROS due to a deficiency in the NADPH oxidase system produce more inflammatory cytokines including IL-1β (88). Since TrxR is the mediator of the NADPH oxidase system, TrxR may link redox-related molecules such as Trx1 and Txnip to NLRP3 inflammasome activation without ROS signaling. Further studies addressing ROS-independent activation of NLRP3 inflammasome will be required to understand the contribution of Trx1/Txnip to NLRP3 inflammasome activation.

Cigarette smoke-driven inflammatory airway disease such as chronic obstructive pulmonary disease (COPD) is aggravated by P2 × 7/inflammasome pathway (89). We reported that Trx1 Tg mice are resistant to COPD induced by cigarette smoke (76, 90, 91). It would be another interesting model for the redoxisome to investigate whether the cigarette smoke-induced inflammation is also regulated by the ratio of Trx1-Txnip interaction and NLRP3-Txnip interaction. The association of Txnip with Trx1 and NLRP3 indicates that Txnip plays a major role in the convergence of multiple signaling pathways that contribute to oxidative stress-related disorders.

Collectively, although Trx1/Txnip is likely involved in the activation of NLRP3 inflammasome, the mechanistic insight is still unclear. Therefore the novel concept of redoxisome consisting of Trx1/Txnip and other redox-related molecules could give us novel insight for the currently unknown mechanism of NLRP3 inflammasome activation in ROS-dependent and-independent manner. Future experiments will have to be performed to establish if the Trx1/Txnip redoxisome system contributes as redox regulatory system to NLRP3 inflammasome activation.

## Thioredoxin1/Txnip, Redoxisome, Cancer Progression, and Other Disease

Txnip overexpression arrests the growth of cells at G0/G1 phase (92), and induces apoptosis in response to various stresses (30, 38, 41, 64, 93). Txnip is reported to inhibit cell cycles by stabilizing p27 via binding to Jab1 (31), and by repressing transcriptional activities via interaction with HDAC1 (94). Moreover Txnip is down-regulated in various human cancer cells and many reports have shown that the down-regulation of Txnip contributes the malignancy of cancer (2). In HTLV-1-infected T cells, the expression level of Txnip is associated with responsiveness to IL-2-dependent growth, and epigenetic silencing of Txnip results in loss of responsiveness to IL-2 (81). In IL-2-independent stage, Txnip silencing loses the sensitivity against glucocorticoid-induced cell death (38). In *in vivo* studies, Txnip has been shown to be a suppressor of the incidence and progression of cancer. The Hcb-19 mice strain with a spontaneous mutation of Txnip gene and Txnip KO mice show the higher incidence of hepatocellular carcinoma (95, 96). Also the Txnip KO mice show earlier onset of *N*-butyl-*N*-(4-hydroxybutyl) nitrosamine (BBN)-induced bladder carcinoma (97). Moreover, in clinical studies, Txnip expression levels decrease following the progression of cancer stages or malignancy in gastric cancer, melanoma, pheochromocytoma, and bladder cancer (97, 98). These results collectively support that Txnip contributes to controlling the malignancy of cancer. It has been also reported that the deficiency of Txnip promotes TNF-α-induced NF-κB activity, that Txnip inhibits mTOR activity by binding Redd1 (99), and that Txnip deficiency enhances the phosphorylation of Akt in response to insulin (28, 72).

Txnip deficiency also enhances phosphorylation of signal transducers. Regarding the relationship between Txnip and cell signaling, phosphorylation of ERK is enhanced in Txnip KO mice bladders during BBN-induced bladder carcinogenesis (97), and TGF-β signaling is enhanced via Smad2 phosphorylation under Txnip-KO or -knockdown condition (100). The loss of Txnip up-regulates a variety of transcriptional activities for several stimuli or ligands, so that deficiency of Txnip could contribute to integrate excessive biological responses and signals for cell survival, malignancy, and the tumorigenesis of cancer. Here, at the mention of the correlation between Txnip and Trx1, many reports show that Txnip interacts with Trx1. Overexpression of Txnip attenuates the reducing activity, and inhibits transcription of Trx1 resulting in increased ROS, which triggers cell cycle arrest or apoptosis (as referred above). The gene expression of Txnip vs. Trx1 shows a reciprocal pattern under the stimulation of vitamin D3 (22), PPAR-γ ligands (26), and suberoylanilide hydroxamic acid (HDAC inhibitor) (92) (Table 1). In the EGF-induced ERK1/2 phosphorylation, Txnip, and Trx1 apparently show reciprocal functions. As shown in several reports, Trx1 overexpression increases activation of ERK1/2 (101, 102) as well as Txnip deficiency. Interestingly, Txnip overexpression strongly reduced Trx1 expression and activity (22, 24, 103), while Txnip deletion has a minor effect of Trx1 expression and activity (104). In regards to TGF-β signaling, TGF-β-induced transcriptional activation is independent of the protein levels of Trx1 (100). These results give us the idea that the event of redox-dependent interactions between Trx1 and Txnip, resulting in the timely attenuation or enhancement of the functions in response to oxidative stresses, may be more biologically important. Further investigation is required to determine whether chronic oxidative stresses cause the inconsistent expression of Trx1 and Txnip by some feedback pathways. Future studies must unveil the physiological significance of the formation of the Trx1/Txnip complex.

**Table 1:** **Reciprocal function between Trx1 and Txnip**.

Biological event (tissue/cell type)	Trx	Txnip	Reference
β-cell apoptosis (islet β-cells)	Protect	Promote	(28, 55–57, 62–65, 67, 68)
Gluconeogenesis (liver)	No report	Activate	(70)
Glucose uptake (muscle, adipose)	No report	Suppress	(28, 59, 71)
Insulin secretion (islet β-cells)	No report	Suppress	(28, 36)
Insulin sensitivity (muscle, adipose)	No report	Suppress	(28, 36, 59, 71, 72)
Inflammation (many cell types)	Suppress	Activate	(29, 75, 78, 82, 84, 89)
NLRP3 inflammasome (macrophage, islet)	Suppress	Activate	(29)
IL-2 responsive (T cell)	Positive	Negative	(80, 81)
Neurodegenerative (brain)	Protect	Promote	(53, 54, 93)
Adiposity/obesity (adipose, liver)	No report	Suppress	(28, 33–36, 72)
ROS production (many cell types)	Reduce	Increase	(20)
Cellular survival (many cell types)	Promote	Inhibit	(19, 20, 42)
Metastasis (many cell types)	No effect	Suppress	(99, 100)
Carcinoma (liver, bladder)	No effect	Suppress	(94–97)
Cardiac hypertrophy (heart)	Protect	Promote	(32)

## A Perspective of Trx/Txnip of Clinical Work

The modulation of cellular redox regulation has emerged as a potential clinical approach for cancer, autoimmune disease, and diabetes. In this review, we proposed the Trx/Txnip signal complex is important for redox regulation and related disorders. The function of Trx is to reduce cellular ROS and related stresses, while Txnip has reciprocal function for Trx. This evidence gives us a novel therapeutic approach to control Trx/Txnip. Since the Trx/Txnip complex acts in various kinds of stresses, Trx/Txnip may form a protein complex with other proteins. In this review, we defined this redox-related signal complex as “Redoxisome” and suggest it may be a promising therapeutic target. Many reports suggest that increasing of Trx1 and decreasing of Txnip expression is beneficial for preventing hyper inflammation, neurodegeneration, and progression of diabetes, while there is the risk to increase the chance of incidents for gastric (105–107), lung (108), and liver (30, 95, 96) cancer, especially with reduction of Txnip expression (Figure 2). To eliminate unwanted side effect such as cancer development, the regulation of Trx1 and Txnip interaction might be a more effective therapeutic approach than the regulating their expression. Further elucidation of the mechanism for redox regulation and pathogenesis of diseases by the Trx/Txnip, redoxisome is required to understand how cells and the whole body integrate the various redox signals under the multiple diseases states and the future findings may give us promising redox regulated therapeutic approach by Trx/Txnip, redoxisome signaling (Figure 2).

## Conflict of Interest Statement

The authors declare that the research was conducted in the absence of any commercial or financial relationships that could be construed as a potential conflict of interest.
